# Long-term effects of endoscopic papillary large balloon dilation in patients with challenging bile duct calculi: A retrospective observational study

**DOI:** 10.1097/MD.0000000000027227

**Published:** 2021-09-10

**Authors:** Hidehiro Kamezaki, Terunao Iwanaga, Takahiro Maeda, Jun-ichi Senoo, Dai Sakamoto, Shin Yasui, Harutoshi Sugiyama, Toshio Tsuyuguchi, Naoya Kato

**Affiliations:** a Department of Gastroenterology, Eastern Chiba Medical Center, Togane, Chiba, Japan; b Department of Gastroenterology, Graduate School of Medicine, Chiba University, Chiba, Japan; c Endoscopy Center, Chiba University Hospital, Chiba, Japan; d Department of Gastroenterology, Chiba Prefectural Sawara Hospital, Katori, Chiba, Japan.

**Keywords:** common bile duct, endoscopic sphincterotomy, gallstones, outcome assessment

## Abstract

Endoscopic papillary large balloon dilation (EPLBD) can be used to treat challenging common bile duct stones. No previous studies have reported intractable cases treated either by EPLBD or mechanical lithotripter use. We aimed to evaluate and compare the long-term effects of EPLBD with mechanical lithotripter use.

This retrospective cohort study reviewed data from 153 patients admitted to the Eastern Chiba Medical Center from April 2014 to March 2020, presenting with common bile duct calculi that could not be removed using a basket or balloon catheter. Patients were divided into groups depending on whether the treatment was performed using a mechanical lithotripter or EPLBD. The primary outcome was the recurrence rate of common bile duct calculi, and the secondary outcome was the rate of postoperative adverse events. The Wilcoxon test was used to compare the 2 groups. Statistical significance was set at *P* < .05.

The median age of patients included in the lithotripter and EPLBD groups were 73 years and 83 years, respectively (*P* = .006), while the sex ratio (male:female) in the groups was 18:13 and 55:67, respectively. The EPLBD group showed a statistically larger median bile duct diameter (13 mm [range: 8–24 mm] vs 11 mm [range: 5–16 mm]; *P* < .001), larger maximal calculus diameter (median, 13.5 mm [range: 8–25 mm] vs 11 mm [range: 7–16 mm]; *P* < .001), and shorter median cumulative treatment time after reaching the duodenal papilla (35.5 minutes [range: 10–176 minutes] vs 47 minutes [range: 22–321 minutes]; *P* = .026) in comparison to the lithotripter group. There was no significant difference in the rate of adverse events between the EPLBD and the mechanical lithotripter groups. The recurrence rate was significantly lower (*P* = .014) in the EPLBD group.

EPLBD increases therapeutic efficacy and reduces treatment duration for patients in whom calculus removal is difficult, without increasing the frequency of adverse events. No serious adverse events were observed. Additionally, EPLBD appears to reduce the risk of long-term recurrence. Future studies are needed to evaluate long-term outcomes in younger patients.

## 1 Introduction

Common bile duct stones, which are often asymptomatic, arise due to the migration of gallstones from the gallbladder into the common bile duct. The prevalence of bile duct stones is often underreported, as most studies detect bile duct stones during cholecystectomy for symptomatic diseases.^[1]^ The prevalence of common bile duct stones in patients with symptomatic gallbladder stones is proposed to be 8% to 18%.^[2]^ The prevalence of bile duct stones, as detected by intra-operative cholangiography, is 4.6% to 12% in Europe and 20.9% in South America.^[3–5]^ A recent systematic review has shown that, among patients with acute cholecystitis, the incidence of common bile duct stones varies from 2.4% to 25%, with an estimated pooled incidence of 13.7%.^[6]^ The most effective and standard treatment for bile duct stones is endoscopic retrograde cholangiopancreatography (ERCP).^[7]^ However, in 10% to 15% of cases, additional techniques are required,^[8]^ typically due to a large stone size (>15 mm) and/or the tapering of the distal bile duct. Endoscopic papillary large balloon dilation (EPLBD), which consists of lithotomy without lithotripsy and dilation of the papilla using a large balloon, was first described by Ersoz et al^[8]^ in 2013 as a technique to address challenging cases. In their study, 58 patients who had an unsuccessful removal of bile duct stones using endoscopic sphincterotomy (EST) and standard basket/balloon extraction proceeded to EPLBD. In 18 patients with tapered distal bile ducts, 89% (n = 16) had successful stone clearance, and in 40 patients with square, barrel-shaped, and/or large (>15 mm) stones, 95% (n = 35) had successful stone clearance. Since this pivotal study, EPLBD has been increasingly used for challenging bile duct stones and is recommended as a treatment option in national and international guidelines.^[1,9]^ Furthermore, EPLBD has been widely described as effective and safe,^[10–15]^ in comparison to EST, in a meta-analysis of 596 patients from 4 randomized controlled trials.^[16]^ Despite the existence of studies comparing the effectiveness of EST compared to EPLBD,^[17–21]^ there have been no studies that have evaluated intractable cases according to whether they proceeded to EPLBD or mechanical lithotripter use. Furthermore, little is known about the long-term effect and prognosis of EPLBD when performed with or without EST,^[22,23]^ and existing data on the long-term outcomes following EPLBD are limited and conflicting.^[17,24–27]^

This study aimed to explore the long-term outcomes associated with EPLBD in our center. We specifically analyzed intractable cases, which we defined as patients in whom the stones could not be removed using a basket and/or balloon.

## 2 Methods

### 2.1 Participants

In this retrospective study, we used a database that contains the clinical data of all patients who underwent ERCP at our center to identify patients for inclusion. A total of 682 patients with choledocholithiasis were treated at the Eastern Chiba Medical Center, a tertiary emergency hospital, during a 6-year period between April 2014 and March 2020, of which 522 patients were treated with only a basket or balloon catheter and 160 had common bile duct calculi that could not be removed using a basket or balloon catheter. Two of these patients experienced malignant biliary stenosis and were excluded; this was the only exclusion criterion of our study. Finally, 158 patients were included in this study. The diagnostic criteria and severity grading of acute cholecystitis were defined according to the Tokyo Guidelines 2013/2018.^[28]^ The patients were divided into a group in which treatment was performed using only the mechanical lithotripter (lithotripter group) and a group in which treatment was performed by EPLBD (EPLBD group). This study was performed in accordance with the Declaration of Helsinki and was approved by the Ethics Committee of the Eastern Chiba Medical Center (approval number: 41). Informed consent was not required because of the retrospective nature of the study.

### 2.2 Equipment

The following equipment was used to perform the procedures described in this manuscript: Extraction Balloon Catheter Plus (Zeon Medical Inc., Tokyo, Japan) and CREPRO Wireguided Biliary Dilatation Balloon Catheter (Boston Scientific Corp., Marlborough, MA); TetraCatchV and FlowerBasketV (Olympus, Tokyo, Japan) basket catheters; Visiglide and Visiglide 2 (Olympus, Tokyo, Japan) guidewires; CleverCut3V (Olympus, Tokyo, Japan) sphincterotome; JF/TJF TYPE 260V duodenoscope (Olympus, Tokyo, Japan); and LithoCrushV mechanical lithotripter (Olympus, Tokyo, Japan).

### 2.3 Procedures

The decision that a stone could not be removed with a basket and/or balloon and that regarding further specific treatment (i.e., EPLBD or lithotripter use) were left to the discretion of the treating clinician. EPLBD was performed as previously described.^[13]^ Midazolam, pentazine, and hydroxyzine were used as sedative and analgesic agents in the periprocedural period. All EPLBD procedures were performed either by a board-certified Fellow of the Japan Gastroenterological Endoscopy Society or by a trainee physician under the direct supervision of a board-certified Fellow of the Japan Gastroenterological Endoscopy Society. If there were no postoperative concerns, the patient commenced oral feeding the day following treatment. After the procedure, all patients were managed in a standard ward setting, and no patients required transfer to intensive care.

In this study, we also included cases in which balloon dilation was less than 12 mm, which is a widely used threshold for defining EPLBD use. There was no absolute minimum cutoff value for patients undergoing dilation to be included in this study. The patients underwent a median dilation of 12 mm (range, 9–18 mm), and 47 patients underwent dilation of less than 12 mm.

### 2.4 Postprocedural follow-up

This was a retrospective study; thus, the follow-up frequency and performance of postprocedural imaging were not standardized.

### 2.5 Outcome measures and data collection

The primary outcome measure in our study was the recurrence rate of common bile duct calculi. The secondary outcome measure was the rate of postoperative adverse events. Our institutional database was used to retrospectively evaluate the patient background, treatment details, adverse events, and recurrence rates. We also calculated and compared the lower bile duct diameter and maximum calculus diameter (both in mm) between the lithotripter and EPLBD groups. Measurements were obtained from the diagnostic cholangiograms.

### 2.6 Statistical analysis

Statistical analyses were performed using SPSS Statistics software (version 24; IBM Japan, Tokyo, Japan). The D’Agostino-Pearson test was performed to assess the normality of the distribution of continuous variables. Comparisons were performed using Pearson chi-squared test for categorical variables and the Mann-Whitney *U* test for continuous variables. Cumulative recurrent common bile duct stone rates were estimated using the Kaplan-Meier method. We used the Gehan-Breslow-Wilcoxon test to investigate the null hypothesis that there is no difference in cumulative recurrence rates among the groups “Lithotripter” and “EPLBD”. Statistical significance was set at a 2-tailed *P* < .05.

## 3 Results

The baseline characteristics of the 2 groups are shown in Table 1. There were 31 patients in the lithotripter group (19.6%), 122 in the EPLBD group (77.2%), and 5 in whom treatment could not be completed (3.2%); thus, data for 153 patients were analyzed. In the lithotripter group, 1 patient in our cohort had a benign biliary stricture due to primary sclerosing cholangitis. Another patient had a recurrence of difficult bile duct stone removal on day 389 after the initial presentation; this patient initially belonged to the lithotripter group, while the recurrence case belonged to the EPLBD group. The EPLBD group comprised 44 patients (36.1%) in whom mechanical lithotripter was also used. The median ages in the lithotripter and EPLBD groups were 73 years (range: 31–96) and 83 years (range: 52–99), respectively, and the median age in the EPLBD group was significantly higher (*P* = .006) (Table 1). No significant differences in sex, disease severity, frequency of postoperative gastric reconstruction, anti-thrombotic agent administration, hypertension, diabetes, heart disease, stroke, kidney disease (estimated glomerular filtration rate < 30), hepatic cirrhosis, or juxtapapillary diverticula were found between the groups. The mean proportion of patients undergoing cholecystectomy after ERCP was significantly higher in the lithotripter group (n = 9/29) than in the EPLBD group (n = 12/104; *P* = .024).

**Table 1:** Overview of the clinical and demographic characteristics in the lithotripter and endoscopic papillary large balloon dilation groups.

	Lithotripter group (n = 31)	EPLBD group (n = 122)	*P* value
Median age, years (range)	73 (31–96)	83 (52–99)	.006
Gender ratio, male:female	18:13	55:67	.196
Disease severity (cholangitis)^∗^ none:mild:moderate:severe	6:12:8:5	27:26:52:17	.174
Previous EST, n	0	18	.049
Frequency of postoperative gastric reconstruction, n (type)	1 (B-1)	5 (B-1, B-1, B-2, B-2, Roux-en-Y)	1.000
Anti-thrombotic agent administration, n	8	35	.750
Hypertension, n	17	66	.941
Diabetes, n	5	29	.361
Heart disease, n	9	31	.682
Stroke, n	4	24	.384
Kidney disease (eGFR < 30), n	2	9	1.000
Hepatic cirrhosis, n	1	2	1.000
Juxtapapillary diverticula, n	14	69	.255
Postcholecystectomy, n	2	18	.354
Cholecystectomy after ERCP, n	9 (out of 29)	12 (out of 104)	.024
Days from ERCP to cholecystectomy, median (range)	51 (22–346)	52 (13–931)	.943

**Figure 1:**
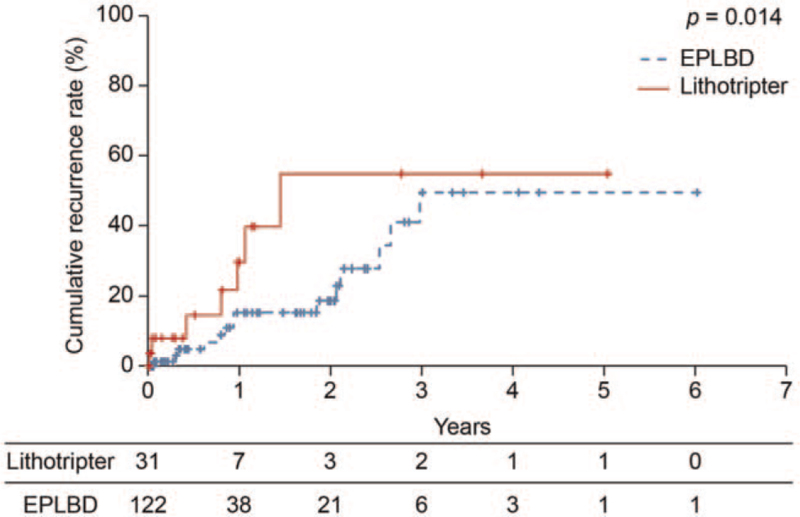
Kaplan-Meier survival analysis comparing the recurrence rate of common bile duct calculi between the EPLBD and lithotripter groups (generalized Wilcoxon test, *P* = .014). EPLBD = endoscopic papillary large balloon dilation.

The median lower bile duct diameters in the lithotripter and EPLBD groups were 11 mm (range: 5–16 mm) and 13 mm (range: 8–24 mm), respectively, and the median maximum calculus diameters were 11 mm (range, 7–16 mm) and 13.5 mm (range: 8–25 mm), respectively, both of which were larger in the EPLBD group (both *P* < .001; Table 2). The number of patients in whom the calculi were completely removed with a single treatment was 9 (29.0%) and 32 (26.2%), respectively, which was not significantly different (*P* = .753). The median cumulative treatment time after reaching the duodenal papilla in the lithotripter and EPLBD groups was 47 minutes (range: 22–321 minutes) and 35.5 minutes (range: 10–176 minutes), respectively, and was shorter in the EPLBD group (*P* = .026). The number of patients in the respective groups with adverse events were as follows (Table 3): pancreatitis, 2 (6.5%) and 7 (5.7%; *P* = 1.000); cholangitis, 2 (6.5%) and 7 (5.7%; *P* = 1.000); hemorrhage, 0 (0%) and 0 (0%); and perforation, 0 (0%) and 0 (0%). All patients recovered from these adverse events with conservative treatment. The recurrence rates after 1, 2, 3, and 4 years were 29.6%, 54.7%, 54.7%, and 54.7% in the lithotripter group, and 15.4%, 18.7%, 49.4%, and 49.4% in the EPLBD group, respectively; the recurrence rate was significantly lower in the EPLBD group (*P* = .014). Figure 1 shows the Kaplan-Meier survival analysis for recurrence rates according to the surgical method used. The mean follow-up time was 65.5 days and 108 days in the EPLBD group and the lithotripter group, respectively.

**Table 2:** Anatomical, pathological, and therapeutic data.

	Lithotripter group (n = 31)	EPLBD group (n = 122)	*P* value
Median lower bile duct diameter, mm (range)	11 (5–16)	13 (8–24)	<.001
Median maximum calculus diameter, mm (range)	11 (7–16)	13.5 (8–25)	<.001
Number of stones, n			.452
- 1, n	9	26	
- 2, n	6	18	
- 3 or more, n	16	78	
No ESTs performed, n	0	5	.562
Calculi completely removed with single treatment, n (%)	9 (29.0)	32 (26.2)	.753
Number of ERCPs required to achieve complete stone removal, n			.406
- 1	9	32	
- 2	15	73	
- 3	6	13	
- 4	0	3	
- 5	1	1	
Median Cumulative treatment time after reaching the duodenal papilla, min (range)	47 (22–321)	35.5 (10–176)	.026

**Table 3:** Adverse events and recurrence in the lithotripter and endoscopic papillary large balloon dilation groups.

	Lithotripter group (n = 31)	EPLBD group (n = 122)	*P* value
Pancreatitis, n (%)	2 (6.5)	7 (5.7)	1.000
Cholangitis, n (%)	2 (6.5)	7 (5.7)	1.000
Hemorrhage, n (%)	0 (–)	0 (–)	–
Perforation, n (%)	0 (–)	0 (–)	–
Recurrence			.014^∗^
- Recurrence at 1 year, %	29.6	15.4	
- Recurrence at 2 years, %	54.7	18.7	
- Recurrence at 3 years, %	54.7	49.4	
- Recurrence at 4 years, %	54.7	49.4	

## 4 Discussion

This study evaluated the long-term effects of EPLBD compared to mechanical lithotripter use in 153 patients recruited from a single Japanese center. The study identified a significantly larger bile duct diameter and larger maximal calculus diameter in the EPLBD group. The cumulative treatment time after reaching the duodenal papilla was shorter in the EPLBD group, and there was no significant difference in the rate of adverse events between the EPLBD and mechanical lithotripter groups. The recurrence rate was lower in the EPLBD group.

The adverse event rate following EPLBD in our study was comparable to that reported in previous studies, with pancreatitis and cholangitis being the most common adverse events reported.^[29]^ It has previously been shown that multiple recurrences of common bile duct stones are not uncommon, with risk factors for recurrence including bile duct size, gallbladder left in situ with stones, and pneumobilia after treatment, and for multiple recurrences, the number of stones identified at the first occurrence.^[30]^ Another study evaluating long-term outcomes over a mean period of 33.7 ± 16.6 months following EPLBD for common bile duct stones found that recurrence occurred in 17% of patients (n = 16), with a large distal common bile duct diameter being the only significant risk factor for recurrence on multivariate analysis.^[26]^ In a recent systematic review regarding the efficacy and safety of common endoscopic methods, EPLBD was evaluated as the safest option.^[31]^ While EPLBD might not be demonstrated to reserve the sphincter function, its long-term outcome was comparable to that of EST.^[27]^ The proportion of patients undergoing cholecystectomy after ERCP was significantly higher in the lithotripter group than in the EPLBD group, and this may be due to a higher proportion of elderly people in the EPLBD group.

The recent MARVELOUS trial^[21]^ has shown that EPLBD without EST is associated with a significantly higher chance of complete stone removal in a single session compared to EST, with no increase in adverse events. The novelty of our study lies in the fact that we compared outcomes in patients with intractable common bile duct stones who underwent EPLBD with those managed using a mechanical lithotripter. Our study serves as a benchmark for future studies addressing this important topic.

Importantly, our study shows that, despite patients undergoing EPLBD with larger bile duct diameters and maximum calculi diameters than those undergoing mechanical lithotripter use, the duration of treatment after reaching the duodenal papilla was shorter and the recurrence rate was lower in the former group. This, combined with the similar adverse event rates observed between the 2 surgical techniques, suggests that EPLBD is a superior treatment modality for intractable cases of common bile duct stones. The recurrence rate in our EPLBD group was higher than that observed in other studies,^[26]^ but this is likely due to the difference in patient characteristics between the studies.

The main limitations of our study include its retrospective nature, the relatively few patients with long-term follow-up data, and the possibility of measurement error associated with the use of diagnostic cholangiography. The latter point is unavoidable as imaging appearances using this modality differ considerably depending on the amount of contrast medium administered to each patient during the scan. In addition, the decision regarding treatment with lithotripsy or EPLBD was left to the discretion of the treating endoscopists and was a potential bias in our study, as it resulted in some differences in the baseline characteristics between the 2 groups. Another limitation of our study was the sample size (n = 153). Finally, in this retrospective study, we could not assess the incidence of late adverse events as a long-term effect of EPLBD. Due to these limitations, the results of our study should be considered indicative rather than conclusive.

In conclusion, our study showed that EPLBD increased the therapeutic efficacy and reduced the treatment time for patients in whom calculus removal was difficult. It did not increase the frequency of adverse events and had no serious adverse events. In addition, EPLBD appears to reduce the risk of long-term recurrence. Future studies should evaluate long-term outcomes in younger patients.

## Acknowledgments

Editorial support, in the form of medical writing, statistical checks, assembling tables, and creating high-resolution images based on authors’ detailed directions, collating author comments, copyediting, fact-checking, and referencing, was provided by Editage, Cactus Communications.

## Author contributions

**Conceptualization:** Hidehiro Kamezaki.

**Formal analysis:** Hidehiro Kamezaki, Terunao Iwanaga.

**Methodology:** Hidehiro Kamezaki.

**Writing – original draft:** Hidehiro Kamezaki.

**Writing – review & editing:** Takahiro Maeda, Jun-ichi Senoo, Dai Sakamoto, Shin Yasui, Harutoshi Sugiyama, Toshio Tsuyuguchi, Naoya Kato.
